# The comorbidity of HIV, hypertension and diabetes: a qualitative study exploring the challenges faced by healthcare providers and patients in selected urban and rural health facilities where the ICDM model is implemented in South Africa

**DOI:** 10.1186/s12913-021-06670-3

**Published:** 2021-07-03

**Authors:** Motlatso Godongwana, Nicole De Wet-Billings, Minja Milovanovic

**Affiliations:** 1 Programme in Demography and Population Studies, University of the Witwatersrand, Schools of Public Health and Social Sciences, Johannesburg, South Africa; 2 Perinatal and HIV Research Unit, Chris Hani Baragwanath Hospital, Johannesburg, South Africa

## Abstract

**Background:**

PLWH are living longer as a result of advancement and adherence to antiretroviral therapy. As the life expectancy of PLWH increases, they are at increased risk of hypertension and diabetes. HIV chronic co-morbidities pose a serious public health concern as they are linked to increased use and need of health services, decreased overall quality of life and increased mortality. While research shows that integrated care approaches applied within primary care settings can significantly reduce hospital admissions and mortality levels among patients with comorbidities, the primary care system in South Africa continues to be challenged with issues about the delivery of quality care.

**Methods:**

This study applied a phenomenological qualitative research design. IDIs were conducted with 24 HCPs and adults living with the comorbidity of HIV and either hypertension or diabetes across two provinces in South Africa. The objective of the research was to understand the challenges faced by HCPs and patients in health facilities where the ICDM model is implemented. The health facilities were purposively sampled. However, the HCPs were recruited through snowballing and the patients through reviewing the facilities’ clinic records for participants who met the study criteria. All participants provided informed consent. The data was collected between March and May 2020. The findings were analysed inductively using thematic content analysis.

**Results:**

The challenges experienced included lack of staff capacity, unclear guidelines on the delivery of integrated care for patients with HIV chronic comorbidities, pill burden, non-disclosure, financial burden, poor knowledge of treatments, relocation of patients and access to treatment. Lack of support and integrated chronic programmes including minimal information regarding the management of HIV chronic comorbidities were other concerns.

**Conclusion:**

The outcomes of the ICDM model need to be strengthened and scaled up to meet the unique health needs and challenges of people living with HIV and other chronic conditions. Strengthening these outcomes includes providing capacity building and training on the delivery of chronic care treatment under the ICDM model, assisted self-management to improve patient responsibility of chronic disease management and strengthening activities for comorbidity health promotion.

## Background

Advancement in antiretroviral therapy (ART) has improved the health outcomes and prolonged the lives of people infected with the human immunodeficiency virus (HIV) [1]. As the life expectancy of people living with HIV (PLWH) increases, they are prone to developing additional chronic diseases [2, 3]. In 2018, the global prevalence of hypertension in PLWH was estimated at 9% and at least 59% of these were living in sub-Saharan Africa [4]. A hospital-based study in Ethiopia found that over 50% of its HIV positive sample reported an additional chronic condition from hypertension, diabetes, cardiovascular disease, Chronic Obstructive Pulmonary Disease (COPD) and cancer [5]. In Zimbabwe, chronic comorbidities among PLHIV are projected to increase by 26% by the year 2035 [6]. Similarly, almost 30% of people living with HIV (PLHIV) in South Africa have at least one other comorbidity [7]. Further research in South Africa shows that about one fifth of PLWH were hypertensive at ART initiation and almost 15% were diagnosed with hypertension at follow up [8].

The deterioration in levels of physical activity and weakness in the metabolic system are some of the factors that increase the vulnerability of developing chronic conditions as people grow older [9]. A national population-based survey in South Africa found that more than 50% of people were not physically active [10]. However, beyond individual physical activity, a study in Swaziland confirms that hypertension in PLHIV is triggered by various mechanisms such as changes in Body Mass Index (BMI), characteristics of the HIV and some ART regimens [11].

Studies in high and low income countries are in agreement that PLHIV are faced with an increased risk of developing hypertension and diabetes [2, 5, 8, 9]. However, the chronic conditions reported by PLHIV may be pre-existing, HIV-related or even due to ageing. For instance, at times, HIV and antiretroviral drugs interact with chronic disease risk factors to lead to the development of chronic diseases [12].

**Fig. 1:**
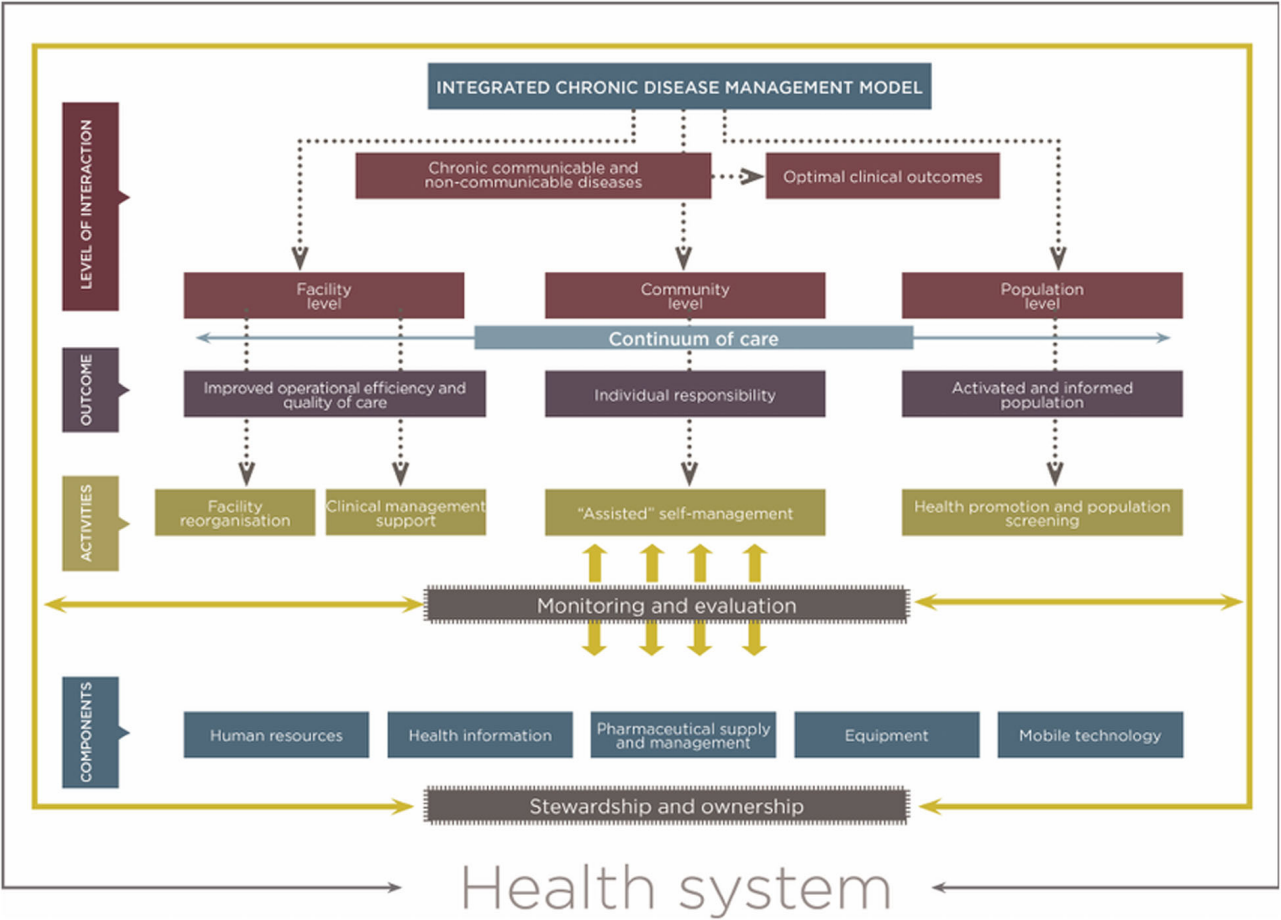
Integrated Chronic Disease Model implemented in South Africa, National Department of Health 2012

Like many countries, South Africa has adopted an Integrated Chronic Diseases Management (ICDM) model which it first introduced in 2011 to address the double burden of HIV and Acquired Immunodeficiency Syndrome (AIDS) and non-communicable diseases [13]. Some of the key outcomes of the model are to provide improved operational efficiency and quality of care ensure patient responsibility and establish an activated and informed population (Fig. 1) that can support individuals living with chronic conditions such as HIV/AIDS and other non-communicable diseases [13].

The health system has also established other avenues to support persons living with chronic comorbidities. For example, initiating adherence clubs that provide support for the life-long treatment of chronic conditions including HIV. In these clubs, a group of about 30 patients who have been on treatment for longer than 12 months meet with a club facilitator (usually a retired healthcare professional or lay counsellor) after every 2 months outside of the mainstream care provided at health facilities to receive their medication, support and motivate one another towards treatment adherence [14].

To achieve the outcomes of the ICDM model, the model proposes reorganising the facility to better manage bookings and patient flow, expanding clinical management support by providing training and guidance on the delivery of the ICDM model, providing assisted self-management through empowering, providing support at the facility and community level and prioritising health promotion and population screening [13]. Some studies have evaluated the implementation of the model and found potential barriers that threaten its success. Mahomed et al. found that adequate training was not provided to nurses and management staff on the implementation of the model. Additionally, he found that resistance towards the adaptation of the model emanated from the perceived increase in workload that would come with integrating chronic services [15]. Other studies have cited barriers to delivering the ICDM model to include overburdened health systems, staff shortages and lack of guidelines on comorbidity care under the model [16]. The objective of this research is to identify the challenges experienced by HCPs and persons living with HIV chronic comorbidities in health facilities where the model is implemented.

Persons diagnosed with the comorbidity of HIV and hypertension or diabetes have unique healthcare needs as compared to persons living with only one of these conditions as they require multiple medicine prescriptions as well as regular monitoring to avoid medicine toxicities and ensure adherence [17]. For some ART compliant patients, treatment fatigue is common and entails coping with side effects, the burden and intensity of treatment as well as taking complicated regimes [18]. Also, structural and institutional barriers to accessing chronic care treatment continue to persist and include poverty, stigma and poor social support [19]. The treatment and management of co-morbidities is difficult for both patients and health providers as they need costly prolonged treatment and care [20]. For health providers, the comorbidity of diseases may be too complicated for simple treatment [21]. For example, patients with chronic comorbidities often require multiple treatment regimens that need to be prescribed and administered carefully to reduce the risk of drug interactions [21].

The increasing prevalence of chronic conditions among PLWH and the unique healthcare needs of this population group require a well-integrated ICDM model. However, the implementation of the model remains suboptimal in many facilities where it is implemented as a result of, among others, health system inefficiencies [22].

After establishing existing barriers to providing comorbid care, some research has called for further studies to understand the experiences of comorbid patients under the CDM model being implemented in South African public healthcare facilities [16]. This study is two-fold in that it investigated the challenges faced by health care providers (HCPs) in delivering the outcomes of the ICDM model, particularly, to patients living with the comorbidity of HIV and hypertension or diabetes as well as provides the perspectives of persons living with these conditions to understand their challenges as well. The study focused on providing an understanding of challenges in terms of the three outcomes of the ICD model (improved operational efficiency and quality care, achieving individual or patient responsibility and an activated and informed population).

## Methods

### Study setting

The study was conducted among participants attending primary health care (PHC) facilities and hospitals in Gauteng and the Eastern Cape (EC) provinces of South Africa. The selection of these two provinces was based on the fact that spatial analysis had shown the EC to be among the provinces with the highest prevalence of chronic comorbidity in South Africa [23]. In this province, the adult HIV prevalence is 25.2%, which is higher than the national average [24]. Gauteng was selected because it comprises the largest (14.7 million) share of the South African population and has the highest (24.0%) percentage of the elderly population, aged 60 years and older, residing in the province [25]. About 17.6% of the adult population in Gauteng is living with HIV [24].

### Study design

We developed semi-structured research tools guided by the three principle outcomes of the ICDM model. For the Key Informant Interviews (KIIs) with healthcare workers, we focused on one of the model outcomes which is to achieve improved operational efficiency and provide quality of care. We developed a set of questions that would help provide information on the barriers to achieving optimal operational efficiency and adequate quality of care. Some of these questions were premised around (1) the treatment and care provided to persons living with HIV and other chronic comorbidities, (2) resource availability and its effect on the delivery of the model, (3) capacity development and training provided to HCPs on HIV chronic comorbidities treatment and care under the model and (4) any other health system challenges that affected efficiency and comorbidity care.

For the in-depth interviews with participants living with HIV and either hypertension or diabetes, we focused on the remaining two outcomes of the ICDM model namely patient responsibility and understanding whether this population group was activated/informed about HIV chronic comorbidities. As a result, we developed questions premised around (1) understanding participants’ knowledge regarding prevention, treatment and management of HIV chronic comorbidities, (2) challenges about treatment access in facilities where the model is implemented, (3) challenges with the self-management of chronic comorbidities and (4) understanding the difficulties with disclosure and social interactions after participants come to the knowledge of the dual diagnosis of HIV and hypertension or diabetes. These research questions were developed to understand the barriers with regards to ensuring patients take responsibility for the management of their comorbid condition and knowledge regarding both the prevention and management of HIV chronic comorbidities.

In this study, we adopted a qualitative phenomenological approach to understanding the experiences shared by both healthcare providers and adults living with HIV comorbidities.

### Study population and data collection procedures

Purposive sampling was used to select 7 PHC facilities and 3 hospitals where the ICDM model is implemented in EC and Gauteng. A combination of clinics and hospitals was used because patients with HIV chronic comorbidities often receive their medical treatment at PHC facilities such as clinics. However, in cases where patient’s conditions are uncontrolled, patient care can be escalated to the hospital level. To be eligible for the study, HCPs were required to have at least 2 years of experience with providing treatment and care to people living with HIV chronic comorbidities.

An independent researcher trained on the study protocol contacted each health facility and explained the study protocol and procedures to the facility manager. Based on the eligibility criteria for HCPs, each facility manager provided the researcher with a list of the contact details for eligible HCPs. To minimise selection bias, the lists were combined and the names of the HCPs were randomised. Then, the sample of HCPs was recruited based on their availability to participate in the research. However, some of the HCPs in the hospitals and clinics could not participate in the study and mentioned that their health facilities were under a lot of pressure given the rise of the Covid-19 pandemic and that the majority of their staff was, in addition to providing health services, preoccupied with Covid-19 training and capacity building. Given this, the study relied on snowballing to recruit HCPs. The HCPs that were successfully interviewed, provided contact details of other HCPs who were not necessarily from the same health facility.

Criteria for patient participants were that one was (1) aged 18 years or above, (2) living with an HIV chronic comorbidity and (3) on ART and receiving treatment at the selected health facilities. The researchers relied on the facility patient records provided by the HCPs/facility manager to identify and generate a list of adult participants that met the study criteria. Recruitment of participants with HIV chronic comorbidities occurred until the point of data saturation.

The study successfully conducted 12 KIIs with healthcare providers and 12 in-depth interviews with participants living with HIV and an additional chronic condition from March to May 2020. At the time of data collection, the Covid-19 pandemic had surged the world and several lockdown restrictions including limitations on movement and face-to-face contact were enforced in South Africa to limit the spread of the virus. Given these circumstances, 17/24 interviews were conducted telephonically and 6/24 were conducted before the implementation of the lockdown regulations.

### Interview process

The HCPs were asked to provide details of adults living with HIV chronic comorbidities in their health facilities using clinic and hospital records. Then, the researchers made telephonic contact with these patients to assess eligibility and obtain participant consent. The objective of the study was explained to all study participants that met the study criteria. For face-to-face interviews, participants provided written consent. For telephonic interviews, the study information sheets were sent via Email and WhatsApp *(as per participant’s preference*) and consent was obtained verbally and audio recorded. The interviews were conducted in English and isiXhosa, a local language in the EC Province*.*

### Data analysis

Where a local language was used, the data was transcribed back to English by an isiXhosa speaking translator and checked for accuracy and completeness. All transcripts were imported into Atlas.ti version 12. The HCP and patient participant interviews were first analysed separately. Analysis began with the HCP interviews, followed by the patient participants. For each study population, the researchers reviewed the findings reported by the participants and developed a set of preliminary codes. We began by using deductive analysis following the ICDM model (Fig. 1). We focused on the three outcomes of the care continuum notably (1) improved operational efficiency and quality of care, (2) patient responsibility and (3) activated and informed population. To provide more depth to the analysis, inductive analysis was conducted to contextualise the research findings. To do this, we analysed the text to identify expressions that shared the same concepts. The respective codes for HCPs and patient participants were analysed and those sharing similarities were merged. We used the final set of codes to develop broader themes. To ensure rigour and trustworthiness of the data, the study firstly used different types of participants (HCPs and adult participants) to understand the challenges with implementation of the outcomes of the ICDM model. Secondly, both face-to-face and telephonic interviews that were conducted yielded similar results. Additionally, the findings from the HCPs were verified against the data from the adult participants to identify results that were congruent or different. In this study, the findings are reported using thematic headings and illustrative quotes.

## Results

A total of 24 interviews were conducted - 12 HCPs and 12 adult participants. Three of the HCPs interviewed were from a rural hospital and clinics in the Eastern Cape, the other three came from clinics in a peri-urban region in the Gauteng province and six HCPs worked in urban clinics across the Eastern Cape and Gauteng provinces. With regards to the adult participants, three were patients in rural clinics and the other three were patients from peri-urban clinics. The remaining six adult study participants were patients across clinics in urban areas in the Eastern Cape and Gauteng. The following themes emerged from the study and are discussed in detail in the sub-sections below (Table 1).

**Table 1:** Emerging themes from the study findings, interviews with HCPs and patients

Theme	Sub-Theme	Illustrative quote
Under-resourced facilities	- Lack of staff - Slow delivery of services - Medication not delivered on time - Poor information provided to patients on chronic disease prevention and management - Lack of HIV and chronic disease support programmes	*“Sometimes the facility itself would be packed meaning that you don’t have enough time to spend with one patient; you have to rush the queue … another thing is that sometimes we have problems with shortage of drugs especially chronic medication like for hypertension and diabetes”,* ***professional nurse, EC****.* *“At the clinic, they didn’t give us enough information about how to live correctly with this condition”,* ***P8 EC.*** *“There is no such programme here that helps people with comorbidities to manage … maybe it’s still coming but not yet”,* ***P2 GP.***
Lack of training and guidance on chronic care delivery	- No provision of training for HCPs on HIV and chronic disease treatment and care - HCPs unavailable to attend training on integrated chronic care	*“To be honest with you, nurses don’t always have the time for refresher courses because most of the time hospitals are short-staffed”,* ***professional nurse, EC.***
Lack of standardisation in adopting the guidelines for chronic care integration		*“HIV falls under the chronic part … so the nurse in chronic will see that client in totality, so she won’t get from chronic to another place [a different department]”* ***professional nurse, EC.*** *“Somehow chronic care is not yet too much integrated into the government sector because I remember asking another patient why she had another date for another treatment … you find one condition [*i.e. *HIV] is handled by the other [referring to one nurse/clinician] and one [other condition* i.e. *hypertension] also handled by the other [a different clinician/nurse]”,* ***Dr, EC.*** *“I get my hypertensive medication and ARVs from two different nurses … I am travelling in between different institutions now because I was transferred to Bara because of my hypertension. And it got controlled, so a month ago, when I went for a check-up, it was down. So, I was transferred back to Bheki Mlangeni for my Anti-Natal Care (ANC) and hypertension. And then, for my ARVs, I go to Chiawelo clinic … I would love to go to one facility for everything”,* ***P12 GP.***
Non-disclosure	- Non-disclosure of HIV status - Stigma - Treatment default	*“It’s disclosure when a patient has not disclosed it becomes difficult for them to cope with two conditions … for example, I am here and I test positive, I have stress, my hypertension becomes uncontrolled whether I am taking treatment or not; the fact that I am stressing is not helping … so disclosure is a problem and it also affects adherence”,* ***professional nurse, GP.*** *“I haven’t told my husband about my illness … there are already problems in our marriage; it will torment us even more”,* ***P7 EC.*** *“There is this cousin sister of mine staying at Jabulani Township; I went there to tell her about what I had found out and she said ooh! as though shocked but then we spoke, she advised me that you are like this, you need to take the treatment. Furthermore, she said I must buy myself food and eat. But then after, I heard that she went around speaking about me behind my back. That hurt me very badly but I told myself, oh well it’s okay; it doesn’t matter … You know what makes a person afraid is that when you tell someone,then, that very someone goes outside and talk about you, it’s how they say it and they laugh”,* ***P2 GP.***
Polypharmacy	- Difficulty with taking more than one treatment - Pill burden	*“They feel like they can be off the medication; they decide which one is more dangerous between HIV and hypertension. Oh! It’s HIV … So they only want to take the treatment for HIV and not the other treatment”,* ***professional nurse, GP.*** *“When you start combining another condition, they become discouraged … I mean I was starting to comply with this one and now you are coming with this other one … when you start introducing something else and adding more pills, they feel like argh! I have lost the battle so why do I have to …*”*,* ***registered nurse, GP.*** *“Well, it is difficult … you should always look at the time; at a certain time, you must take the pills … For example, I must take pills for BP then at night, I must take pills for HIV every day at 20:00. So, it’s that thing that the time must always be on mind and guard that I don’t drink pills late or early”,* ***P4 GP.***
Poor knowledge of treatments	- Names of treatment taken - Understanding of chronic medication	*“90% of patients do not know their treatment … The hypertensive and DM patients … they don’t know their treatment … its now easier with ARVs because there is regimen one and regimen two so we found an easier way of asking them by saying if they take one tablet at night or two, that is, in the morning and evening … which is the difference between regimen one and two,* ***Dr. EC.*** *“They don’t understand the term chronic; they think that after they have taken treatment for a month and then they come for a check-up … you will tell them that they are fine and stable then they stop taking medication”,* ***professional nurse, GP.*** *“I don’t know their name; I just drink pills”,* ***P9 EC.***
Relocation of patients	- Moving from clinic to clinic	*“Most of our patients are taking medication where they are not staying; the people we are serving are not from this area. Most of them … they are from another community”,* ***professional nurse, GP.***
Socio-economic issues	- Access to health care - Long travel distance to the hospital	“*Some patients will tell you that I don’t have food at home and don’t even have money to go fetch that treatment at the hospital so mostly it’s social factors”,* ***Dr. EC.*** *“The clinic is far; worse, there are no forms of transportation here. We are in rural areas. It is not a walkable distance and I have a leg problem”,* ***P10 EC.***

### Health care provider characteristics

The sample of HCPs included 1 registered nurse, 9 professional nurses and 2 doctors across three hospitals and seven clinics. With regards to gender, the HCPs comprised eleven females and one male. All HCPs had spent at least 2 years in their profession and in providing treatment and care to PLWH and/or chronic conditions. We asked the providers to detail the types of chronic conditions that they commonly attended to among PLWH. All HCPs cited hypertension and/or diabetes as the most prevalent chronic conditions to affect adults diagnosed with HIV. Of the twelve HCPs interviewed, all mentioned that most of their HIV positive adult patients had hypertension while only 6 mentioned that both hypertension and diabetes were prevalent among adults living with HIV.

### Characteristics of patient participants

Of the 12 HIV positive participants, 10 were diagnosed with hypertension and two with diabetes. All were on ART (Table 2).

**Table 2:** Characteristics of adults living with HIV Comorbidity (Hypertension & Diabetes)

AGE	Frequency (N)	Percentage (%)
30–35	2	16.66
36–40	2	16.66
41–45	1	8.33
46–50	4	33.33
51–55	2	16.66
56–60	1	8.33
**SEX**		
Female	11	91.66
Male	1	8.33
**MARITAL STATUS**		
Single	7	58.33
Married	3	25
Divorced/Separated	2	16.66
**LEVEL OF EDUCATION**		
Primary	3	25
Secondary	8	66.6
Matric	1	8.33
**EMPLOYMENT STATUS**		
Employed	6	50
Unemployed	6	50

The majority (33%) of the participants were aged between 46 and 50, mostly female (92%) and almost 60% reported that they were single. In terms of socio-economic status, more than two-thirds had received secondary education. There was an equal balance between the number of participants that were employed and those that were unemployed.

### Improved operational efficiency and quality of care

Overall, the HCPs cited three main challenges pertaining to providing efficient and quality care to patients living with the comorbidity of HIV and hypertension or diabetes under the ICDM model.

#### Under-resourced facilities

Most healthcare providers reported that their health facilities were under-resourced in terms of staff capacity. The nurses cited being frequently overwhelmed with the patient flow. Some said that they sometimes did not take their lunch breaks or reduce them to 15 min. Others reported that they took their lunch while consulting with patients. The fatigue resulting from missing lunch breaks and/or the workload placed on providers to attend to as many patients seems costly for holistic patient care. One nurse shared the following experience:

*“Sometimes the facility itself would be packed meaning that you don’t have enough time to spend with one patient; you have to rush the queue … another thing is that sometimes we have problems with shortage of drugs especially chronic medication like for hypertension and diabetes”, professional nurse, EC.*

The patient participants echoed that the lack of staffing in healthcare facilities was responsible for the many notable inefficiencies such as the slow delivery of services. One patient said: *“To us, it seems as though they are just slow but they are not slow; it’s just that they are short-staffed”, P2 GP.*

In the nurse’s shared experience, concern was raised over the lack of on-time delivery of chronic medications particularly in the rural areas. This hinders efforts to achieve improved operational efficiency because it means patients may need to return on a different date to receive their medication. This could be one of the reasons health facilities and HCPs become overworked on specific days. Further, the issue of delayed delivery of medication risks patients defaulting from treatment.

#### Lack of training and guidance on chronic care delivery

Two major concerns were raised by healthcare providers with regards to receiving adequate training on the delivery of the ICDM model. Firstly, because of under-resourced facilities, some HCPs reported that they were overworked to find time to attend training and when they attended, they were not as focused during lessons because they were exhausted. For example, one HCP said:

*“To be honest with you, nurses don’t always have the time for refresher courses because most of the time hospitals and clinics are short-staffed”, professional nurse, EC*

In the second account, HCPs who had moved between facilities or different departments reported that they had some experience with providing treatment and care for HIV, hypertension and diabetes respectively. However, they did not receive training on dealing with HIV chronic comorbidities. One nurse summarised her experience as follows:

*“Let me be honest with you, when I was working at XXX clinic, I had just come from working in a hospital, so at the hospital, I was working at medical and didn’t know much about what is going on in the primary health care and I did not get training like specific to comorbidities … I was just thrown in the deep end”, professional/triage nurse, EC.*

#### Lack of standardisation in adopting the guidelines for chronic care integration

An important objective of the ICDM model is to provide integrated chronic disease treatment and care. Providing clear guidelines and training to HCPs on how chronic treatment and care should be provided under the model is a key factor to achieve success with the implementation of the model. However, as alluded to earlier, several HCPs mentioned that they did not receive training on implementation of the model and the study findings reveal that while the existing model guidelines are clear, they are not standardised across all facilities that have adopted the model.

For example, some nurses reported that they provided holistic treatment for all chronic conditions including HIV, hypertension and/or diabetes. In their account, a patient with HIV and hypertension has one file, one appointment card and can receive their chronic medication from the same nurse or during the same clinic visit. However, other nurses said that some of their patients had different clinic dates for HIV and hypertension or diabetes treatment. These findings come to show that different health facilities are operating differently in terms of providing integrated chronic treatment. When HIV and other chronic conditions are treated and cared for differently by HCPs, it holds potential to affect the continuity of care for persons living with comorbidities because, for example, patients may feel tedious to attend the clinic on different dates for different conditions. They can even think that they are being treated differently by the health system for having certain health conditions. A doctor in one of the hospitals mentioned that, at times, a patient diagnosed with HIV and either hypertension or diabetes would have each condition attended to separately:

*“Somehow chronic care is not yet too much integrated into the government sector because I remember asking another patient why she had another date for another treatment … you find one condition [i.e. HIV] is handled by the other [referring to one nurse/clinician] and one [other condition i.e. hypertension] also handled by the other [a different clinician/nurse]”, Dr, EC.*

Interestingly, when the adult participants were asked about their experiences of receiving integrated chronic care and the quality of the care provided, we found that patients were happy with the attitude of healthcare providers in providing treatment and care:

“*Every time when I come here, they don’t just give me my medication and say here is your medication go, they will ask me this and that; how are the pills treating you, how does your body feel and so on”, P4 GP.*

However, we found that routine testing for high blood pressure was provided but not for diabetes. With the exception of one patient (who was pregnant at the time of interview), most reported receiving their HIV and chronic care treatment from one nurse within the same facility. However, some patients raised concerns about the separation of queues for patients receiving HIV treatment and those receiving care for other conditions and the slow delivery of services:

*“I had a problem with the way the queues are formed. People with HIV had their line and other people who came to the clinic for different issues had their lines … that puzzled me a lot”,* ***P10 EC.***

One of the advantages of the ICDM is to ensure the non-segregation of patients. Although patients were not asked further on the reasons they thought the separation of queues for HIV and chronic care was taking place in their health facilities, the separation of queues in health facilities can perpetuate stigma in health facilities because it would make it easy for patients to identify who is being managed for HIV.

### Patient responsibility

The study explored providers’ and patients’ perspectives of the role of patients to adhere to treatment and ensure adequate management of their conditions. HCPs mentioned that the self-management of diseases was difficult for patients and that this was evident in the rate of default from HIV treatment and subsequently from other chronic medication as well. The HCPs elaborated on several reasons and experiences that cause patients to discontinue treatment, which we have organised under sub-themes as below. These themes include non-disclosure, polypharmacy (the use of multiple medications [26]), poor knowledge of treatments, constant movement of patients and socio-economic issues.

#### Non-disclosure

The study found that non-disclosure and the resulting consequences were a concern for HCPs. Many HCPs mentioned that some of their patients had not disclosed their HIV status to either their partners, family, peers and/or employers. The reasons for non-disclosure included stigma and fear of discrimination. One of the consequences of non-disclosure is treatment default. The HCPs reported that patients that had not disclosed their HIV or chronic status often defaulted from treatment. All providers agreed that HIV was the most difficult to disclose compared to other chronic conditions such as hypertension or diabetes. However, even after disclosure of HIV status, some HCPs were to the view that most of their patients found it difficult to disclose their other chronic conditions because it was too many illnesses for anyone to understand. One of the nurses said:

*“People don’t want to disclose I think it’s because of the stigma or whatever … they will even tell you that even the family haven’t accepted that they are HIV positive and now there’s hypertension or diabetes so sometimes it’s difficult … they are even too scared to disclose the status to their partners, parents and when not disclosing their status, adherence is going to fall”, registered nurse, GP.*

There were different accounts from the patient participants. Some agreed with the HCPs that it was much easier to inform people that they were hypertensive or diabetic as opposed to telling them about their HIV status. Others related their non-disclosure as a fear of discrimination, stigma or judgement. Two of the participants shared their experiences as follows:

*“ … . you know I was once employed as a house helper and my employer found me taking my pills and she fired me from work just for that … and I have worked for them for many years but I was fired for that … Now it is many of us at work who are taking medications and we all know each other’s clinic appointment dates but our employers are not aware of that. So, when one goes for their clinic appointment ,you just tell them about the high blood condition but not the HIV one”, P5 GP.*

*“Well, yes, people do gossip about me because I lost weight; my body was not like this … many are not aware of my high blood pressure condition but they are aware of the HIV one because I am losing weight … the discrimination that I am receiving is mainly because of the HIV but not the high blood”, P4 GP.*

#### Polypharmacy

In this study, the major problem with PLWH and other chronic conditions is polypharmacy for as they suffer multiple diseases, they need to take multiple medications. The HCPs mentioned that another leading cause for treatment default was because PLWH and additional chronic conditions were subjected to taking different medications, sometimes taking many pills at single or different times. They reported that this often led to treatment fatigue or patients weighing which of the treatments between HIV or hypertension they thought was more important or had the least side effects:

*“I think pill burden is a problem for patients … for hypertension, it depends on how your high blood is elevated, for example, I can start you on one drug or two or three drugs … so the fact that you have three drugs on top of the ARV drug … now taking four tablets in a day it’s a lot for them. So, you find that some of them take the HIV drug only and say no to the hypertension one; I will see it later”, professional nurse, GP.*

Indeed, some patients reported treatment fatigue whereby they became tired of taking multiple medications. In some cases, the patients reported that they were fatigued from the side effects of some of the medications and wished that they were taking medication for only one condition. Two of the patients said the following:

*“They are five … I take two for HIV and three for high blood … hey! It is very hard … they are a lot and stressful to take … worse when I hear or see other people that are taking 1 pill for HIV and probably less for chronic … it worries me a lot, I just wonder what is so unique about my illnesses”, P8 EC.*

*“I wish I only drank ARVs … These pills (high blood) sometimes make me feel dizzy; I don’t know if maybe I drink it at the wrong time or what; I become so dizzy that I have decided to only drink it when I have a headache. I no longer drink it the way I am supposed to … I think it’s better maybe if they didn’t give us pills but rather the medication for high blood as in form of an injection; when you come, they just inject you.”, P3 GP.*

#### Poor knowledge of treatments

We found that participants had poor knowledge of their HIV and chronic medication with most being unable to name the medications that they were using apart from saying it was for HIV and or hypertension/diabetes. The HCPs mentioned that it was important for patients to know the names of their treatments to ensure that in cases where the patient relocates, he/she can tell their next health provider what treatment they were on. This is so because some of the HCPs said that some of their patients relocate often and would transfer to other health facilities without requesting a transfer letter. Additionally, the HCPs said it was not enough for patients to just identify that they were on HIV or diabetes or hypertension medication because each condition had different regimens depending on the complexity of the patient’s condition. In the healthcare provider’s account:

*“Most of the time patients don’t even know the names of the tablets that they are taking”, professional nurse, EC.*

This corroborated the experiences of the patient participant with one saying:

*“I don’t know their name; I just drink pills”, P9 EC.*

However, the patients mentioned that they were ignorant of the importance of knowing the names of their medications.

#### Relocation of patients and non-request for clinic transfer letters

Healthcare providers raised concern over the constant movement of patients from one geographical area to the next. They mentioned that patients that intend to relocate do not request their local clinic to provide them with transfer letters to allow the next clinic to know the client’s medical history:

*Our clients are moving a lo;, today they will be here and the next day, they are in KZN … we tell clients that when they leave, they should come and we give them a transfer letter but some do not follow that … usually, what they do is that when they come to an institution, they will say that it is my first time because the client knows that she is supposed to bring a transfer letter”, professional nurse, EC..*

#### Socio-economic issues

Patient responsibility is difficult to achieve especially for patients with a low socio-economic status. In most of the PHC included in this study, the patients were unemployed and experienced several financial challenges that affected access to health facilities, food security and their ability to take treatment accordingly. One healthcare provider reported that:

*“Some patients will tell you that I don’t have food at home and don’t even have money to go fetch that treatment at the hospital so mostly its social factors”, Dr, EC.*

Similarly, the patient participant agreed that unemployment was one of the factors that made the self-management of chronic conditions difficult. One participant went on to say:

*“I am unemployed. For someone to be living with these conditions and on the other hand not working is a problem … because even those pills, you cannot just drink them when you have not yet eaten anything; you must eat before you can be able to drink pills … you must eat fruits and other things; all those things require money because even with this condition, we hear from doctors or nurse’s advice that it is very important that we always eat lots of fruits and that requires money”, P4 GP.*

### Activated and informed population

The third outcome of the ICDM model is to achieve an activated and informed population. According to the model, health facilities should prioritise informing the population on preventing and managing chronic conditions including HIV. The availability of technology is essential for knowledge transfer and the communication between health facilities and patients. When patients are well informed, they are better able to treat and manage their health conditions. HCPs were asked about their role in ensuring patients with HIV comorbidities were informed and supported about the prevention and management of chronic comorbidities. Providers reported that support and information were provided through different structures. These included community healthcare providers and social workers who provide health talks and support through platforms such as WhatsApp and adherence clubs. They mentioned that these support initiatives were implemented by organisations not affiliated to the clinics but often non-governmental organisations under donor funding:

*“We have a WhatsApp group for most of them to give them an allowance to ask whatever question from me or the counsellors … also, there are clubs for people that are taking the treatment … the clubs are not only for people who are HIV positive;, they are for everyone who is chronic but stable and they don’t have to see a professional now and then … when I say stable, I mean the blood pressure is controlled”, professional nurse, GP.*

*“We get support from NGOs … they come to the clinic because the government doesn’t have staff … and they assist with things like tracing defaulting patients”, professional nurse, EC.*

However, the study reports on a different perspective from the patient participants. Most patients reported social workers or support groups were unavailable to provide information and/or support to patients living with HIV chronic comorbidities. Others reported that they had not received sufficient information about managing their conditions from HCPs as well. For example, two of the participants mentioned the following:

*“We just get our medication and go home … we do not have support programmes that help us to cope with mental stress for having these conditions”, P9 EC.*

*“I did not get much information about HIV and ways of preventing vulnerability from other chronic diseases; the information I got was very vague … they only said when you are diagnosed with HIV, it’s easy to get other diseases but that time I didn’t have diabetes or knew it existed”, P7 EC.*

### Rural-urban differences and similarities in challenges

In this study, the challenges experienced by both HCPs and the adult participants were similar across both the rural and urban areas where the study was conducted. A few exceptions were with regards to the ease of accessibility to health facilities and the degree of stigma experienced by participants in rural areas.

For instance, across both provinces, the sample of HIV positive adult participants had poor knowledge regarding the prevention and management of hypertension or diabetes:

*“I did not get much information about HIV and ways of preventing vulnerability from other chronic diseases; the information I got was very vague … they only said when you are diagnosed with HIV, it’s easy to get other diseases but that time I didn’t have diabetes or knew it existed”, P7 EC.*

*“I didn’t know it’s (hypertension) preventable or could be prevented … at the HIV section, they told us not to have sex with a man without using a condom...P3, GP’.*

The study findings revealed that poverty and unemployment were a challenge for patients in both rural and urban areas. For patients affected by these socio-economic issues, the self-management of HIV chronic comorbidities was difficult because they did not always have money to buy food to take their medication or to afford a healthy diet. This has made treatment adherence difficult to manage for HCPs alike. For example, one HCP said:

*“I am serving in a community that is faced with poverty. So, sometimes, it’s hard for them to take medication due to lack of food”, professional nurse, GP”.*

In this study, another issue affecting treatment adherence was the stigma, which was reported on by the adult participants and HCPs in both the rural and urban areas sampled. However, the degree of stigma reported on was increased for participants in rural areas as compared to those in urban areas. Health information and information on current affairs were slow to reach some rural communities. As such, some participants mentioned that their communities were ill-informed on issues regarding HIV and comorbidities, which perpetrated existing stigma around living with HIV and other illnesses. However, in more urban communities, access to media platforms such as televisions, radios and social media made people better understand and address stigma around HIV patients.

“It’s still hard to talk about it in this community … I don’t know whether we’re illiterate or that we still have stigma”, P9 EC.

Finally, while accessibility to healthcare was a challenge to patients in rural areas because of the long travel distance to the clinics, all of the adult participants and HCPs from peri-urban or urban health facilities reported that access to treatment was convenient. However, across both provinces, some participants complained about the lack of HIV and chronic disease support programmes to assist diagnosed patients to jointly manage living with HIV chronic comorbidities.

## Discussion

Several challenges continue to exist with delivering the three outcomes of the ICDM model. In this study, these included under-resourced facilities and poor adaption of the guidelines on the delivery of the model. These findings are consistent with research by Mahomed et al. conducted on the sustainability of the model, which found an inability of HCPs to easily adapt to the model and the lack of support from clinical management to implement the model that had an impact on the outcomes of the ICDM model [27]. About patient participants, they had concerns over non-disclosure, polypharmacy, socio-economic challenges and the lack of support programmes for managing HIV chronic comorbidities that affect the ability of PLWH and other chronic diseases to receive integrated chronic care at the PHC level.

### Improved operational efficiency and quality of care

According to the ICDM, providing quality of care in health care facilities is dependent on improvement in operational efficiency [28]. In this study, HCPs across both the EC and Gauteng province mentioned several operational inefficiencies that can potentially have an impact on the treatment and care provided to patients with HIV chronic comorbidities. An important one was the lack of training on guidelines for integrated chronic care delivery for HIV comorbid patients. For example, one of the HCPs said:

“*I did not get training like specific to comorbidities … I was just thrown in the deep end”, professional/triage nurse, EC.*

Inadequate training and the unavailability of guidelines have been reported as some of the challenges for the unsuccessful implementation of the ICDM model in other studies as well [29, 30]. In this study, as with related studies, the challenges included limited staff capacity coupled with overburdened health facilities and inconsistency in the adaptation of chronic care integration guidelines across the different health facilities [13].

In this study, the experiences of adults living with HIV chronic comorbidities corroborate these operational deficiencies in the health system. The reported experiences from patients regarding slow service delivery and the divide in queues for HIV treatment versus treatment for other chronic conditions were concerning. Health system inefficiencies such as lack of staff and unclear guidelines for the care of patients with multi-morbidities threaten the successful implementation of the ICDM [16]. Research suggests that current budgeted costs for implementing the ICDM model are sufficient, however, minimal additional costs are required for improving patient flow, managing bookings and the training of staff [31].

One of the objectives of the model was to use the HIV programme to scale up services for chronic conditions [28]. In this study, we found that routine testing for chronic conditions was done only for hypertension. The improvement in routine testing for hypertension was unexpected given that other studies have reported on the malfunctioning of blood pressure equipment and medicine stock-outs for hypertension at PHC facilities [22, 32]. A recent audit of the quality of care for diabetes patients in PHC facilities in South Africa showed that the ability to provide comprehensive care to diabetic patients was compromised by the lack of resources [33]. In this study, we found that, apart from hypertension, patients were not screened for diabetes or other chronic conditions at the PHC level.

Some empirical evidence has suggested that operational inefficiency negatively influences patient satisfaction [34]. However, results in this study showed that adult patients with the comorbidity of HIV and hypertension or diabetes were satisfied with the attitude of HCP in providing treatment and care. We found that most adults reported that HCP was supportive and remained professional despite the healthcare deficiencies.

### Individual/patient responsibility

Health care providers reflected on the difficulty of patients to take responsibility for managing their conditions noting that this was evident in their experiences with patients that default from HIV treatment and, subsequently, from other chronic medications. According to providers, the management of the disease is a challenge to patients because of issues of non-disclosure, polypharmacy, poor knowledge of treatments, constant movement of patients and socio-economic issues. However, research by Mahomed et al. found that PHC facilities continue to focus on curative treatment and care thereby neglecting the need to emphasise prevention and health promotion such that patients are better able to take responsibility for their health [15]. The management of diseases is expected to be difficult when patients are not informed or supported at the facility and community level [28]. However, it was interesting to find that HCPs and social workers provide support to patients through health talks at the health facilities, social media platforms and adherence clubs.

Indeed, patients diagnosed with HIV comorbidities in this study found the self-management of comorbidities difficult. For example, diabetes requires self-management outside of the health facility whereby patients continuously monitor their glucose levels and maintain a healthy diet [16]. Further challenges include stigma against persons living with HIV comorbidities, pill burden, financial difficulties, challenges with maintaining a healthy lifestyle and access to treatment.

The patients interviewed in this study reported being unsatisfied with the information they receive regarding the prevention and management of HIV chronic comorbidities. Some complained about the existence of stigma at a community level which made them fearful of disclosing their HIV status in particular. The implications of stigma on health-seeking behaviour and access to healthcare have been researched widely. Findings from Turan et al. showed perceived community stigma was associated with poor treatment adherence among PLWH [35]. A qualitative study on the experiences of stigma among people on ART in Tanzania also found that stigma made disclosure difficult and undermined ART adherence and social support [33]. Similar to other studies, the HIV stigma reported in this study came in the form of gossiping, judgment and lack of support [34]. Reports of existing stigma and discrimination at the community level also suggest an uninformed population. For example, research in South Africa has found that lack of education and communities that were less knowledgeable about HIV/AIDS were more stigmatising compared to those whose population was educated [36].

### Activated and informed population

Access to healthcare in South Africa remains a challenge particularly in rural areas because of the distance between the patients’ home and health facility which often involves travelling costs [32]. Our study supports these findings as we found that health facilities in the rural areas were not within close proximities resulting in patients having to walk long distances and some patients being unable to fetch their treatments because of the travelling costs.

This study found a discordant account of the existence of support given by healthcare providers and patients. Contrary to the report given by HCPs, the adult participants mentioned that support from the facility and community was minimal. They reported receiving minimal information about managing HIV chronic comorbidities and the absence of support groups. The study acknowledges that ensuring an activated and informed population may be a costly component of the ICDM model [29]. However, health systems need to be transparent about the difficulties in delivering this component. The scale up of this component can improve treatment adherence and the prevention and management of HIV chronic comorbidities [29].

## Conclusion

The three outcomes of the ICDM model (*improved operational efficiency and quality of care, patient responsibility an activated and informed population*) need to be strengthened to meet the unique health needs and challenges of people living with HIV and other chronic conditions such as hypertension and diabetes. To strengthen these outcomes, the activities outlined in the model should be scaled up. Facility reorganisation activities can request for additional clinical support to provide better management of patients flow and redistribute some of the ICDM implementation costs towards providing support groups and assisted self-management to improve patient responsibility of chronic disease management. It provides capacity building and training on the delivery of chronic care treatment under the ICDM model ensures that guidelines on the integration of chronic diseases such as HIV, hypertension and diabetes are standardised across all health facilities where the model is implemented. Lastly, it scales up health promotion at the population, community and individual level.

Overall, the described activities to strengthen and scale up the outcomes of the ICDM model require the national health department(s) to invest and better manage the allocation and distribution of resources at the PHC level. Furthermore, given the challenges currently present with the implementation of the model, activities adopted at the PHC to provide integrated chronic treatment need to be continuosly monitored and evaluated against the desired outcomes of the model.

## Limitations

This study has important limitations. Firstly, we did not evaluate how long the ICDM model had been implemented in each of the health facilities that were sampled in this study. As a result, the suboptimal outcomes of the ICDM model mentioned in this study may reflect facilities that had recently adopted the model. Secondly, the findings in this study are representative of patients and healthcare providers in only two selected provinces in South Africa. Lastly, given that the study was conducted during the Covid-19 pandemic and during lockdown restrictions, the findings should be interpreted with consideration to the limitations in the data collection methods. Nonetheless, the results presented in this study are essential for policies that aim to improve the adherence and the quality of care received by patients living with HIV and the comorbidity of hypertension or diabetes.

## Data Availability

The data that support the findings of this study are available from Motlatso Godongwana but restrictions apply to the availability of these data, which were used under license for the current study and so are not publicly available. However, data are available from the authors upon reasonable request and with permission of Motlatso Godongwana.
